# NMA plus antitumor therapy versus antitumor therapy alone in patients with BCLC stage B or C hepatocellular carcinoma and pre-cachexia or cachexia: protocol for a prospective, multicenter, randomized, open-label phase II trial

**DOI:** 10.3389/fgstr.2026.1926294

**Published:** 2026-09-29

**Authors:** Ruihong Dai, Facheng Lu, Weibing Leng, Qing He, Wenhao Guo, Tao Hai, Chang Liu

**Affiliations:** Interventional Diagnosis and Treatment Center, West China Hospital, Sichuan University, Chengdu, Sichuan, China

**Keywords:** cachexia, hepatocellular carcinoma, nanocrystalline megestrol acetate, pre-cachexia, randomized controlled trial, study protocol

## Abstract

**Background:**

Cancer anorexia–cachexia syndrome (CACS) is prevalent among patients with Barcelona Clinic Liver Cancer (BCLC) stage B or C hepatocellular carcinoma (HCC) and is strongly associated with poor prognosis, reduced treatment tolerance, and impaired quality of life. Megestrol acetate (MA) has demonstrated efficacy in improving appetite and body weight; however, conventional formulations are limited by variable bioavailability. This trial will evaluate the efficacy and safety of nanocrystalline megestrol acetate (NMA) in patients with BCLC stage B or C HCC and pre-cachexia or cachexia receiving systemic therapy combined with transarterial chemoembolization (TACE) or hepatic arterial infusion chemotherapy (HAIC).

**Methods:**

This multicenter, randomized, open-label, parallel-group Phase II trial protocol will enroll 68 adults aged 18–75 years with BCLC stage B or C HCC and pre-cachexia or cachexia. Participants will be stratified according to BCLC stage and weight loss during the previous 6 months and then randomized in a 1:1 ratio to receive either NMA (625 mg/day for 12 weeks) plus antitumor therapy (systemic therapy combined with transarterial chemoembolization [TACE] or hepatic arterial infusion chemotherapy [HAIC]) or antitumor therapy alone. Antitumor therapy will comprise systemic therapy combined with TACE or HAIC. The co-primary endpoints are the proportions of Anorexia/Cachexia Subscale-12 (A/CS-12) responders and body mass index (BMI) responders at Week 12. Secondary endpoints include objective response rate, progression-free survival, changes in the L3 skeletal muscle index (L3 SMI), and the incidence of adverse events. Exploratory endpoints will evaluate inflammatory and nutritional biomarkers as well as quality of life, assessed using the European Organisation for Research and Treatment of Cancer Quality of Life Questionnaire-C30 (EORTC QLQ-C30).

**Discussion:**

This trial will provide preliminary comparative data on NMA plus antitumor therapy versus antitumor therapy alone in patients with BCLC stage B or C HCC and pre-cachexia or cachexia.

**Ethics:**

The study protocol was approved by the Ethics Committee of West China Hospital, Sichuan University (IRB No. 2025-2539). The trial is registered at ClinicalTrials.gov NCT07356037.

## 1 Introduction

Cancer anorexia–cachexia syndrome (CACS) is a complex metabolic syndrome primarily characterized by progressive weight loss and the selective depletion of skeletal muscle, with or without loss of adipose tissue (1). CACS is closely associated with shortened survival, an increased incidence of treatment-related adverse events, reduced adherence to anticancer therapy, and a marked deterioration in quality of life (2, 3). Liver cancer was the third leading cause of cancer death worldwide, and hepatocellular carcinoma (HCC) is its predominant histologic type (4). CACS is highly prevalent among patients with HCC, with approximately 33.3% presenting with a pre-cachexia stage at diagnosis and 23.7% in the cachexia stage (5). Patients with HCC complicated by CACS are less likely to receive systemic or locoregional anticancer therapies and experience significantly poorer survival outcomes (6, 7). According to the Barcelona Clinic Liver Cancer (BCLC) staging system, the prevalence of CACS among patients with stage B and C HCC is 23.5% and 34.7%, respectively (5). For these patients, locoregional approaches, such as transarterial chemoembolization (TACE) or hepatic arterial infusion chemotherapy (HAIC), in combination with systemic therapies—including chemotherapy, targeted therapy, or immune checkpoint inhibitors—may enhance tumor control and prolong survival (8–10). However, patients who experience weight loss during treatment generally have a poorer prognosis (11, 12).

Pharmacological treatment of CACS mainly includes progestogens, corticosteroids, and other anti-inflammatory agents (13). Among these options, megestrol acetate (MA) is widely recognized as a key therapeutic agent for CACS because of its well-documented effects on appetite stimulation and weight gain (14–17). However, conventional MA is classified as a Biopharmaceutics Classification System (BCS) class II drug, and its oral bioavailability is strongly influenced by food intake. Systemic exposure to conventional MA is lower under fasting than fed conditions, which may contribute to variability in drug exposure (18–20). In contrast, the nanocrystalline formulation of MA is an oral suspension designed to reduce food-dependent variability in absorption. In pharmacokinetic studies, NMA 625 mg and conventional MA 800 mg showed comparable exposure under fed conditions, whereas NMA produced greater exposure under fasting conditions. These findings do not establish clinical dose equivalence (21).

Nanocrystalline megestrol acetate (NMA) has been associated with improvements in body weight and appetite in patients with CACS (22, 23). However, its potential benefit in patients with BCLC stage B or C HCC and pre-cachexia or cachexia who are planned to receive combined systemic and interventional antitumor therapy remains unclear. This prospective, randomized, open-label Phase II trial aims to evaluate the efficacy and safety of NMA plus antitumor therapy versus antitumor therapy alone in patients with BCLC stage B or C HCC and pre-cachexia or cachexia who are scheduled to receive systemic therapy combined with TACE or HAIC.

## 2 Methods

### 2.1 Study overview

This study is a prospective, randomized, multicenter, open-label, parallel-group Phase II clinical trial protocol designed to evaluate the efficacy and safety of NMA in combination with antitumor therapy (systemic therapy plus interventional therapy), compared with antitumor therapy alone, in improving appetite and body mass index (BMI) in patients with BCLC stage B or C HCC and pre-cachexia or cachexia. The enrollment period will run from March 2026 to March 2028, followed by a 12-month follow-up period commencing after the enrollment of the last participant. The study design and assessment schedule are detailed in **Figure 1** and **Table 1**, respectively.

**Figure 1:**
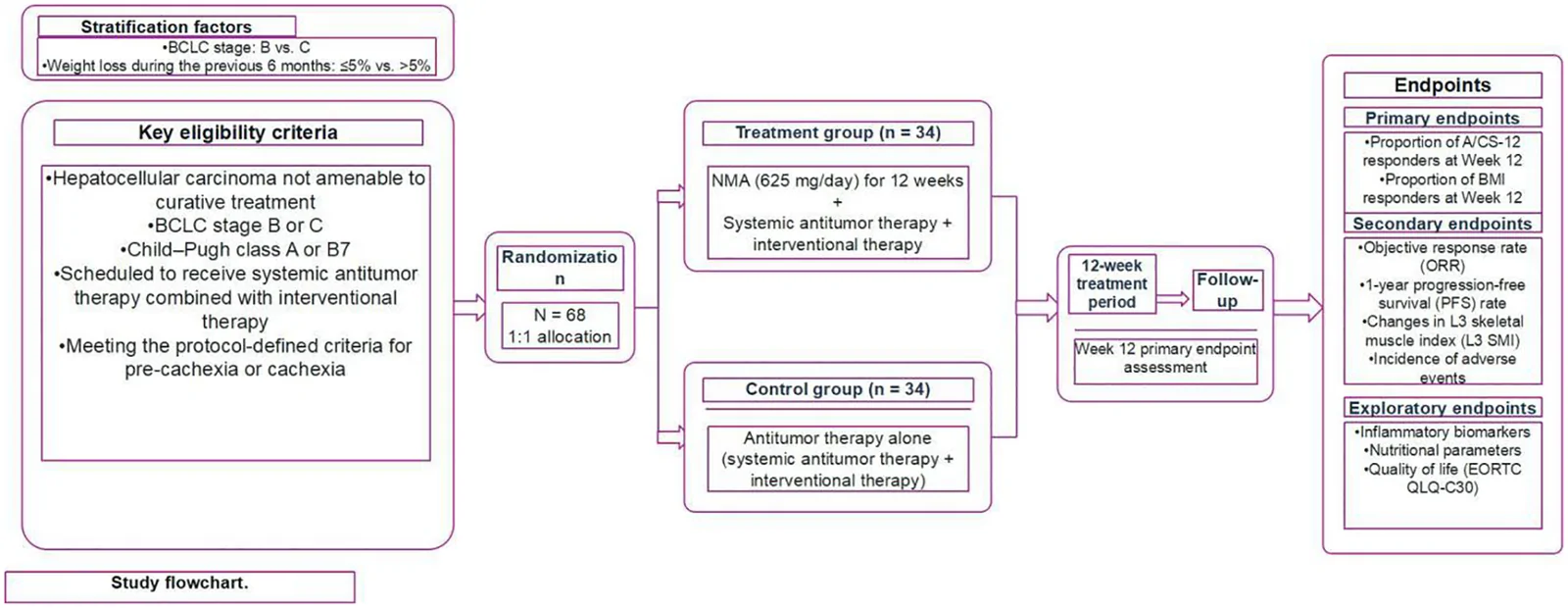
Study flowchart.

**Table 1:** Schedule of study assessments.

Assessments	Pre-enrollment baseline (≤7 days)	Treatment period (week 4)	Treatment period (week 8)	Treatment period (week 12)	Safety follow-up, 30 ± 7 days after the last dose	Long-term follow-up
Enrollment: eligibility screen and Informed consent^1^	✓					
Pathological information, when available^2^						
Baseline characteristics^2^	✓					
Laboratory Tests^2^	✓	✓	✓	✓	✓	✓
Imaging examination^2^	✓	✓	✓	✓	✓	✓
NMA^3^		✓	✓	✓		
antitumor therapy^3^		✓	✓	✓		
Toxicity/Adverse Event monitoring	✓	✓	✓	✓	✓	✓
body mass index	✓	✓	✓	✓	✓	✓
Quality of Life^4^	✓	✓	✓	✓	✓	✓
Appetite^4^	✓	✓	✓	✓	✓	✓
Survival		✓	✓	✓	✓	✓

1. The criteria for screening eligibility can be found in the subsequent section on patient selection.

2. Specific items should refer to the subsequent data collection.

3. NMA and systemic antitumor therapy will be administered in 3-week treatment cycles. NMA will be given for 4 cycles, totaling 12 weeks of treatment. Interventional therapy (TACE or HAIC) will be administered at the investigator’s discretion according to the patient’s treatment needs.

4. Appetite will be assessed using the A/CS-12, and quality of life will be assessed using the EORTC QLQ-C30.

Participants and treating investigators will not be blinded because no placebo will be used. Imaging and body-composition assessors and statisticians will be blinded to treatment allocation.

This protocol follows the SPIRIT guidelines, and a completed checklist will be provided as **Supplementary Material**.

### 2.2 Participant eligibility

Eligible participants must meet the following key inclusion criteria: (1) HCC not amenable to curative treatment; (2) BCLC stage B or C; (3) Child–Pugh class A or B7; (4) fulfillment of the protocol-defined criteria for pre-cachexia or cachexia listed in **Table 2**; (5) age between 18 and 75 years; (6) an Eastern Cooperative Oncology Group (ECOG) performance status of 0–1; and (7) an estimated life expectancy of ≥6 months. Full eligibility criteria, including the organ-function thresholds, are listed in **Table 2**.

**Table 2:** Study eligibility criteria.

Inclusion criteria	Exclusion criteria
1. Voluntarily signed written informed consent form (ICF).	1. Tumor invasion of the main trunk of the portal vein.
2. Aged 18–75 years at the time of enrollment.	2. Presence of any condition affecting gastrointestinal absorption, such as dysphagia, malabsorption, or uncontrollable vomiting; receiving enteral or parenteral nutrition; anorexia due to neurological disorders, psychiatric illness, or pain that makes eating difficult.
3. Eastern Cooperative Oncology Group (ECOG) performance status of 0–1.	3. Currently taking or planning to take other medications that increase appetite or body weight, such as corticosteroids (excluding short-term dexamethasone during chemotherapy), androgens, progestins, thalidomide, olanzapine, anamorelin, or other appetite stimulants.
4. Expected survival ≥ 6 months.	4. Patients with Cushing’s syndrome, adrenal or pituitary insufficiency; patients with poorly controlled diabetes.
5. Histologically or cytologically confirmed hepatocellular carcinoma not amenable to or unsuitable for curative surgical resection, as assessed by the investigator; no prior systemic therapy; Barcelona Clinic Liver Cancer (BCLC) stage B–C.	5.Postmenopausal women with abnormal vaginal bleeding within one year; premenopausal women with abnormal endometrial thickening (>15 mm) within one year.
6. Child–Pugh class A or B7.	6. Active autoimmune disease requiring systemic treatment within the past two years (e.g., treatment with disease-modifying drugs, corticosteroids, or immunosuppressants). History of non-infectious pneumonia/interstitial lung disease requiring systemic corticosteroid treatment, or current non-infectious pneumonia.
7. Planned to receive interventional therapy in combination with systemic antitumor therapy.	7.Presence of uncontrolled comorbidities, including but not limited to decompensated liver cirrhosis, renal failure, uncontrolled metabolic disorders, severe active peptic ulcer or gastritis, or psychiatric/social conditions that may limit compliance with study requirements or the ability to provide written informed consent.
8. At least one measurable tumor lesion according to mRECIST.	8. Within 6 months prior to first dose: any arterial thromboembolic events, venous thromboembolic events of grade 3 or above (per NCI CTCAE v5.0) requiring urgent medical intervention, such as pulmonary embolism or intracardiac thrombus; transient ischemic attack, cerebrovascular accident, hypertensive crisis, or hypertensive encephalopathy. Within 1 month prior to first dose: acute exacerbation of chronic obstructive pulmonary disease. Current hypertension with systolic ≥160 mmHg or diastolic ≥100 mmHg despite oral antihypertensive therapy.
9. Meeting the protocol-defined criteria for pre-cachexia or cachexia Pre-cachexia diagnostic criteria (all of the following must be met): 1. Anorexia; 2. Non-volitional weight loss ≤ 5%; 3. Systemic inflammation (CRP > 5 mg/L). Cachexia diagnostic criteria: Any one of the following, in combination with anorexia or systemic inflammation (CRP >5 mg/L): 1. Non-volitional weight loss > 5% within 6 months; 2. BMI < 18.5 kg/m² and non-volitional weight loss > 2% within 6 months.	9. Severe infection within 4 weeks prior to first dose, including but not limited to infections requiring hospitalization, sepsis, or severe pneumonia; active infection treated with systemic anti-infective therapy within 2 weeks prior to first dose (excluding antiviral therapy for hepatitis B or C).
10. Adequate organ function, defined as follows: Absolute neutrophil count ≥1.5 ×10^9^/L, platelet count ≥100 ×10^9^/L, and hemoglobin ≥90 g/L, without blood-component transfusion or hematopoietic growth-factor support within 7 days before treatment; Calculated creatinine clearance ≥50 mL/min using the Cockcroft–Gault formula; Urine protein ≤1+ or 24-hour urinary protein <1.0 g; Total bilirubin ≤1.5 × the upper limit of normal, or ≤3 × the upper limit of normal in patients with liver metastases or suspected Gilbert syndrome; AST and ALT ≤2.5 × the upper limit of normal, or ≤5 × the upper limit of normal in patients with liver metastases; Serum albumin ≥28 g/L; INR and activated partial thromboplastin time ≤1.5 × the upper limit of normal, unless the participant is receiving anticoagulant therapy and the values are within the expected therapeutic range; Left ventricular ejection fraction ≥50%.	10. Any past or current disease, treatment, or laboratory abnormality that may confound study results, affect full participation in the study, or make participation not in the patient’s best interest.

Patients receiving enteral or parenteral nutrition are excluded because these interventions may directly affect appetite and body-weight endpoints. Patients with Child–Pugh B7 are eligible only if all protocol-specified organ-function criteria are met.

### 2.3 Treatment

Enrolled participants will be stratified by tumor stage (BCLC B vs. C) and the percentage of weight loss within the past 6 months (≤5% vs. >5%). A centralized stratified block-randomization system will be used. An independent statistician not involved in trial conduct will generate the allocation sequence using R. Block size will be determined by the statistician and concealed from the investigators. Participants will be randomized 1:1 to NMA plus antitumor therapy or antitumor therapy alone.

- Treatment group: NMA + antitumor therapy
- Control group: Antitumor therapy alone

NMA and systemic antitumor therapy will be administered in 3-week treatment cycles. In the treatment arm, NMA oral suspension (125 mg/mL) will be given orally at 5 mL per day (625 mg/day) for four cycles, totaling 12 weeks of treatment. The study drug will be continued until disease progression or for a maximum of 12 weeks. Dose adjustments and temporary interruptions will be permitted as clinically indicated.

Antitumor therapy will consist of systemic therapy—such as tyrosine kinase inhibitors, antiangiogenic agents, immune checkpoint inhibitors, or other protocol-permitted regimens—combined with HAIC, conventional TACE, or drug-eluting bead TACE. All treatments will be administered in strict accordance with clinical practice guidelines and the respective prescribing information.

Site investigators will monitor and manage AEs and report relevant safety information to the Ethics Committee. Monitoring will focus on thromboembolic events, adrenal suppression, hyperglycemia, edema, and hormonal effects. Participants will be informed of their right to withdraw from the study at any time without any consequences.

### 2.4 Study discontinuation criteria

The trial treatment must be discontinued immediately under any of the following circumstances:

1. An adverse event (AE) that meets the protocol-defined criteria for permanent discontinuation of the study drug, or any condition that, in the investigator’s judgment, contraindicates further administration;
2. Participant request to discontinue treatment;
3. Initiation of a new antitumor therapy, including the use of another investigational agent;
4. Loss to follow-up;
5. Participant death;
6. Significant protocol deviation (e.g., poor treatment adherence, withdrawal for justified reasons, pregnancy or planned pregnancy, or failure to meet essential study requirements);
7. Investigator decision to terminate treatment;
8. Radiologically confirmed disease progression according to mRECIST;
9. Clinical disease progression in the absence of radiologic confirmation;
10. Any other condition deemed appropriate by the investigator.

### 2.5 Data collection

1. Baseline Patient Characteristics

 • Age (years)
 • Sex (male/female)
 • BMI, kg/m²
 • History of smoking, alcohol intake, and medication use
 • Comorbidities
 • ECOG performance status
 • Child–Pugh classification
 • Hepatitis B virus (HBV) status
 • Hepatitis C virus (HCV) status

2. Laboratory Assessments

 • Alpha-fetoprotein (AFP),
 • Carbohydrate antigen 19-9 (CA19-9)
 • Carcinoembryonic antigen (CEA, ng/mL)
 • Hemoglobin (g/dL)
 • White blood cell count (×10^9^/L)
 • Absolute neutrophil count (×10^9^/L)
 • Platelet count (×10^9^/L)
 • Glomerular filtration rate (GFR, mL/min)
 • Aspartate aminotransferase (AST, U/L)
 • Alanine aminotransferase (ALT, U/L)
 • Total bilirubin (μmol/L)
 • Creatinine (μmol/L)
 • Thyroid function indices

3. Imaging Assessments

 • Liver magnetic resonance imaging (MRI) or computed tomography (CT)
 • Chest CT scans
 • Bone scintigraphy
 • Liver ultrasound
 • Electrocardiogram (ECG) findings

4. Tumor Characteristics

 • Maximum tumor diameter (cm)
 • Tumor differentiation, when available
 • Tumor staging according to the AJCC 8th edition TNM classification

5. Nutritional and Quality-of-Life Assessment

 • Appetite assessment using the Anorexia/Cachexia Subscale-12 (A/CS-12)
 • Lean body mass assessment based on the L3 SMI
 • Quality-of-life assessment using the European Organisation for Research and Treatment of Cancer Quality of Life Questionnaire-C30 (EORTC QLQ-C30)

Body weight will be measured at each scheduled visit using a calibrated scale under standardized conditions. Measurements will be obtained, where feasible, at approximately the same time of day before breakfast, after voiding, and with shoes and outer clothing removed. For participants with ascites, estimated dry weight will be calculated by reducing measured body weight by 5%, 10%, or 15% for mild, moderate, or severe ascites, respectively. A further 5% will be deducted for bilateral pedal edema. The reductions will be combined when both ascites and bilateral pedal edema are present. BMI will be calculated from estimated dry weight when applicable.

L3 SMI will be measured on a single axial CT or MRI image at the third lumbar vertebral level. The psoas, erector spinae, quadratus lumborum, transversus abdominis, and external and internal oblique muscles will be identified and quantified. Total skeletal muscle area will be measured using slice-O-matic or ImageJ and divided by height squared. Whenever possible, the same imaging modality will be used for each participant throughout the study.

Any nutritional counselling, oral nutritional supplements, physical-activity interventions, or other supportive care provided during the study will be recorded.

### 2.6 Outcome measures

1. Primary Endpoints

 • Proportion of A/CS-12 responders at Week 12. An A/CS-12 responder is a participant with an increase of ≥4 points from baseline in the A/CS-12 total score at Week 12.
 • Proportion of BMI responders at Week 12. A BMI responder is a participant with an increase in BMI from baseline at Week 12 (ΔBMI >0 kg/m²).

2. Secondary Endpoints

 • Objective response rate assessed according to mRECIST
 • 1-year progression-free survival (PFS) rate
 • Changes in L3 SMI
 • Distribution of treatment cycles and incidence of dose reductions
 • Incidence of adverse events (AEs)

3. Exploratory Endpoints

 • Changes in inflammatory markers, including C-reactive protein (CRP), interleukin-1 (IL-1), interleukin-6 (IL-6), interferon-γ (IFN-γ), and tumor necrosis factor-α (TNF-α)
 • Changes in nutritional parameters, including albumin and hemoglobin
 • Improvement in quality of life, assessed using the EORTC QLQ-C30

### 2.7 Sample size calculation

The sample-size calculation was based on the two co-primary endpoints. Because no external trial fully matched the study population, control group, and endpoint definitions, the response-rate assumptions were based on previous MA and NMA studies and investigator judgment.

Patients will be randomized in a 1:1 ratio. The calculation used a normal approximation for two independent proportions with unpooled variances and no continuity correction, a one-sided α of 0.025, and 80% power. Approximately 25 evaluable participants per group are required for appetite-response rates of 50% versus 15%, and 29 per group for BMI-response rates of 40% versus 10%.

After allowing for 15% attrition, 34 participants will be enrolled per group, for a total of 68. This is expected to yield approximately 29 evaluable participants per group.

A fixed-sequence procedure will control the family-wise type I error. Appetite response will be tested first at a one-sided α of 0.025. BMI response will be tested at the same level only if the appetite endpoint is statistically significant; otherwise, formal testing will stop.

### 2.8 Data management and monitoring plan

Study data will be collected by trained personnel using validated Case Report Forms (CRFs) to ensure accuracy, completeness, and timeliness, and processed independently by two data entry teams. No independent data and safety monitoring committee has been established for this study. All CRFs will be securely archived for at least five years with read-only access to prevent *post-hoc* modifications.

### 2.9 Statistical analysis

The intention-to-treat population will include all randomized participants and will be used for the primary efficacy analyses. The full analysis set will include participants who receive at least one study treatment and have a baseline body-weight measurement. The safety set will include all participants who receive at least one study treatment and will be analyzed according to the treatment actually received. The per-protocol population will be a subset of the full analysis set excluding participants with major protocol deviations prespecified before database lock and will be used for supportive analyses.

For the co-primary endpoints—the proportion of patients achieving appetite improvement (A/CS-12) and the proportion with BMI increase during treatment—exact two-sided 95% confidence intervals (CIs) will be calculated using the Clopper–Pearson method. Treatment groups will be compared using a Cochran–Mantel–Haenszel test stratified by BCLC stage (B vs. C) and percentage weight loss during the previous 6 months (≤5% vs. >5%). The fixed-sequence testing procedure described in the Sample Size Calculation section will be applied. The secondary endpoint, ORR, will be analyzed similarly. PFS will be estimated using the Kaplan–Meier method, including the 1-year PFS rate. The median PFS and its two-sided 95% confidence interval will be calculated using the Brookmeyer–Crowley method with log-log transformation if estimable. Between-group PFS will be compared using a stratified log-rank test, and the hazard ratio and its two-sided 95% CI will be estimated using a stratified Cox proportional hazards model. Additional adjustment for study center and systemic antitumor therapy will be explored if estimable.

Week 12 will be the primary assessment time point for both co-primary endpoints. A/CS-12 and BMI measurements obtained at other scheduled visits will be analyzed supportively. Missing A/CS-12 and BMI data will be handled using multiple imputation under a missing-at-random assumption, with complete-case analyses performed as sensitivity analyses. No imputation will be performed for missing quality-of-life or imaging data, which will be analyzed using available observations.

Changes from baseline in nutritional and quality-of-life parameters will be summarized descriptively. Mixed-effects models, adjusted for baseline scores and time since randomization, will be employed to compare treatment groups with respect to L3 SMI, A/CS-12, and EORTC QLQ-C30 scores.

All statistical analyses will be conducted using SPSS v22 or R v4.3.1. The fixed-sequence procedure described above will be used for the co-primary endpoints; other statistical tests will be two-sided, with p<0.05 considered statistically significant.

### 2.10 Confidentiality

All study-related data will be retained at the study site under secure conditions. Participants’ personal identifiers will be protected using encrypted, access-controlled data systems. For confidentiality purposes, all study materials—including laboratory findings, questionnaire responses, analysis datasets, and administrative records—will be labeled only with unique, anonymized study identification codes.

## 3 Discussion

CACS is a multifactorial condition marked by persistent skeletal muscle loss, with or without adipose tissue depletion, which is not fully reversible with conventional nutritional support and leads to functional impairment (24). Management of appetite and body weight in CACS patients is therefore clinically important. Notably, patients with anorexia exhibit significantly poorer survival than those without anorexia, with the largest reported difference in median overall survival reaching 56.21 months (25). A BMI-adjusted weight grading system has demonstrated that both body weight status and the degree of weight loss are associated with patient survival. In this system, BMI was categorized into five groups (≤20.0, 20.0–21.9, 22.0–24.9, 25.0–27.9, and ≥28.0 kg/m²), and percentage weight loss (%WL) was classified into five levels (2.5%–5.9%, 6.0%–10.9%, 11.0%–14.9%, ≥15.0%, and weight stable [≤ 2.4%]). Using a 5 × 5 matrix analysis, patients who were weight stable (≤2.4%) with a BMI ≥25.0 kg/m² (grade 0) had the longest median survival (20.9 months; 95% CI, 17.9–23.9). Survival decreased progressively with lower BMI and higher %WL, reaching 14.6 months (grade 1; 95% CI, 12.9–16.2), 10.8 months (grade 2; 95% CI, 9.7–11.9), 7.6 months (grade 3; 95% CI, 7.0–8.2), and 4.3 months (grade 4; 95% CI, 4.1–4.6) (26). In patients with HCC, the incidence of anorexia is reported to be 50%–60% among those undergoing TACE treatment (27, 28). Furthermore, approximately 35% of HCC patients receiving systemic targeted therapy develop cachexia, which has been recognized as an independent adverse prognostic factor for both OS and PFS (7). Evidence also suggests that nutritional intervention may be beneficial across the cachexia continuum, particularly during pre-cachexia and cachexia, when earlier intervention may offer greater therapeutic potential (29). Accordingly, the present study will enroll patients with pre-cachexia or cachexia and HCC who are scheduled to receive HAIC or TACE in combination with systemic antitumor therapy.

Progestational agents can suppress inflammatory cytokines through both humoral and neuroregulatory mechanisms, thereby enhancing appetite and promoting weight gain (14–17). Dose–response effects of megestrol acetate have been reported. In a randomized trial, patients with cancer cachexia received megestrol acetate at 160, 480, 800, or 1,280 mg/day for 3 months. The greatest improvements in appetite and body weight were observed with 800 mg/day (30). A pilot study compared concentrated megestrol acetate suspension with conventional suspension in patients with HIV-associated unintended weight loss (20, 31). In addition, a prospective real-world study conducted in China compared NMA (625 mg/day) with the conventional formulation (800 mg/day) administered continuously for 12 weeks. At week 12, the NMA group achieved a mean weight gain of 4.49 kg versus 2.10 kg in the conventional formulation group (mean difference, 2.39 kg; P < 0.001). Patients receiving NMA also exhibited significantly greater improvements in anorexia (P < 0.001), nausea and vomiting (P = 0.002), and overall health status (P < 0.001) (22). Nonetheless, whether NMA provides clinical benefit in patients with BCLC stage B or C HCC and pre-cachexia or cachexia who receive combined systemic and interventional antitumor therapy remains unclear. The present Phase II trial is designed to address this evidence gap.

The present study has several limitations. First, the antitumor treatment regimen is not fully standardized, and heterogeneity in systemic and interventional treatments may affect efficacy outcomes, particularly PFS. Second, the open-label, non-placebo-controlled design may introduce performance and reporting bias, particularly for patient-reported appetite and quality-of-life outcomes. No universally accepted pharmacological standard has been established for cancer cachexia; therefore, antitumor therapy alone was selected as the control rather than another cachexia-directed drug. Third, the modest sample size limits the power of subgroup analyses. Finally, BMI may be affected by ascites or fluid retention in patients with HCC and should therefore be interpreted together with ascites status and L3 SMI.

## Data Availability

The raw data supporting the conclusions of this article will be made available by the authors, without undue reservation.
